# Pharmacists’ role in harm reduction: a survey assessment of Kentucky community pharmacists’ willingness to participate in syringe/needle exchange

**DOI:** 10.1186/s12954-018-0211-4

**Published:** 2018-01-25

**Authors:** Amie Goodin, Amanda Fallin-Bennett, Traci Green, Patricia R. Freeman

**Affiliations:** 1 College of Pharmacy, Pharmaceutical Outcomes and Policy, University of Florida, 1225 Center Drive, HPNP 2320, Gainesville, FL 32610 USA; 2 College of Nursing, University of Kentucky, Lexington, KY USA; 3 Emergency Medicine, Injury Prevention Research Center, College of Medicine, Brown University, Providence, RI USA; 4 Pharmacy Practice and Science, Center for the Advancement of Pharmacy Practice, College of Pharmacy, University of Kentucky, Lexington, KY USA

**Keywords:** Needle exchange, Syringe exchange, Harm reduction, Community pharmacy practice, Injection drug use, Prevention

## Abstract

**Background:**

Pharmacists’ role in harm reduction is expanding in many states, yet there are limited data on pharmacists’ willingness to participate in harm reduction activities. This study assessed community pharmacists’ willingness to participate in one harm reduction initiative: syringe/needle exchange.

**Methods:**

In 2015, all Kentucky pharmacists with active licenses were emailed a survey that examined attitudes towards participation in syringe/needle exchange. Response frequencies were calculated for community pharmacist respondents. Ordinal logistic regression estimated the impact of community pharmacist characteristics and attitudes on willingness to provide clean needles/syringes to people who inject drugs and to dispose of used syringes/needles, where both dependent variables were defined as Likert-type questions on a scale of 1 (not at all willing) to 6 (very willing).

**Results:**

Of 4699 practicing Kentucky pharmacists, 1282 pharmacists responded (response rate = 27.3%); the majority (*n* = 827) were community pharmacists. Community pharmacists were divided on willingness to provide clean needles/syringes, with 39.1% not willing (score 1 or 2 of 6) and 30% very willing (score 5 or 6 of 6). Few were willing to dispose of used needles/syringes, with only 18.7% willing. Community pharmacists who agreed that pharmacists could have significant public health impact by providing access to clean needles expressed 3.56 times more willingness to provide clean needles (95% CI 3.06–4.15), and 2.04 times more willingness to dispose of used needles (95% CI 1.77–2.35). Chain/supermarket pharmacists (*n* = 485, 58.6% of community pharmacies) were 39% less likely to express willingness to dispose of used needles (95% CI 0.43–0.87) when compared with independent community pharmacists (*n* = 342, 41.4% of community pharmacies). Independent pharmacists reported different barriers (workflow) than their chain/supermarket pharmacist colleagues (concerns of clientele).

**Conclusions:**

Kentucky community pharmacists were more willing to provide clean needles than to dispose of used needles. Strategies to mitigate barriers to participation in syringe/needle exchange are warranted.

**Electronic supplementary material:**

The online version of this article (10.1186/s12954-018-0211-4) contains supplementary material, which is available to authorized users.

## Background

Syringe and needle exchange programs (NEPs) are a well-established intervention to reduce the transmission of HIV among people who inject drugs (PWID) [1]. Further, NEPs are a safe space for clients to form trusted relationships with outreach workers or healthcare providers [2, 3]. NEPs can also serve as a mechanism for PWID to access a range of other health prevention services such as hepatitis and tuberculosis screening, adult vaccinations, [4] wound care, [5], and overdose response training [5, 6].

Harm reduction activities, including NEPs for PWID, have been led by public health communities for decades in the USA. The pharmacists’ role in harm reduction is being expanded in several states as part of the effort to combat the spread of infectious disease and increases in overdose mortality [7]. Though NEPs have been shown to be both effective [8] and cost-effective [9] interventions for disease prevention in previous studies, they remain politically controversial [10] in the general populace, manifesting as inconsistencies in federal and state funding for NEP services [11].

A 2017 meta-analysis of 14 evaluations of pharmacy-based NEPs concluded that pharmacy-based programs are effective interventions in reducing risk behaviors among PWID, yet in the USA, few such programs exist [12]. As an alternative to authorizing true NEPs, many states have expanded access to clean needles and syringes by authorizing the sale of syringes and needles without a prescription and have provided syringe-related exemptions in state drug paraphernalia laws. As of 2016, only five states prohibited the non-prescription sale of syringes and needles [13]. However, Kentucky, similar to many other states, requires the pharmacist to record names and addresses of individuals purchasing syringes, as well as the syringes’ intended use. This requirement may undermine patient trust and places the pharmacist in conflict with state drug paraphernalia laws if the pharmacist perceives the syringes are to be used to administer illicit drugs.

The Centers for Disease Control and Prevention reports that Kentucky, Tennessee, and West Virginia have experienced the greatest surge in incidence of acute hepatitis B infections of all states, with an increase of 114% in new hepatitis B cases from 2009 to 2013, while incidence in other states remained stable during this time period [14]. Kentucky and surrounding states have also experienced a 364% increase in incidence of hepatitis C infections between 2006 and 2012 compared with relatively stable incidence of hepatitis C in other states during this time period [15]. The increase in hepatitis B and C infections, along with an increase in observed incidence of HIV transmission in neighboring Indiana during this time period, [16] has been driven by injection drug use of heroin and prescription opioids. These explosive increases in the transmission of blood-borne viruses recently observed in Kentucky and surrounding states have fueled legislative efforts for harm reduction. Kentucky passed legislation in March 2015 granting municipalities the authority to implement NEPs and providing exemptions for these programs from Kentucky’s drug paraphernalia laws [17]. Non-medical injection users of prescription opioids in other states often access needles and syringes from pharmacies, [18–20] which presents an opportunity for harm reduction interventions in community pharmacy settings if they, too, could be granted a similar drug paraphernalia law exemption. However, there is little information available in the literature about pharmacists’ changing perceptions of these programs after the recent increase in overdose mortality and rise in injection-driven infections, so the potential for widespread implementation in Kentucky remains unclear. The objective of this study was to assess community pharmacists’ willingness to participate in a NEP and to gauge attitudes and perceptions of community pharmacists’ role in harm reduction efforts related to overdose and their role in public health.

## Methods

A survey instrument was developed to assess pharmacist overall perception and attitudes about the pharmacists’ role in harm reduction activities with regard to injection drug use and opioid overdose, as well as willingness to participate in select elements of NEPs, namely, providing clean needles and syringes to PWID and disposing of used syringes and needles in their pharmacy. A team of practicing pharmacists and pharmaceutical policy researchers developed the survey questions and adapted question sets from previous surveys [21, 22] and then partnered with Kentucky Board of Pharmacy (BOP) for assistance in survey distribution and promotion to pharmacists. The survey instrument is available as Supplementary Material (see Additional file 1—Survey instrument).

The BOP distributed an email containing a cover letter with an explanation of the study and a link to the electronic survey to all pharmacists licensed to practice in Kentucky on June 23, 2015. Respondents were screened for eligibility prior to the beginning of the survey by affirming active Kentucky pharmacist licensure. Those who did not affirm licensure were exited from the survey. Survey data were collected in the web-based application Research Electronic Data Capture (REDCap) [23], and no identifying information or linkages to either respondent email addresses nor IP addresses were recorded with survey responses.

A reminder email was delivered to all pharmacists 1 week following the initial survey invitation, and a final reminder and call to participation was distributed via email 2 weeks following initial contact. Two weeks after the final email reminder, the survey link was deactivated and data collection was stopped. The University of Kentucky Institutional Review Board approved the survey protocol.

### Variables and statistical analysis

Response frequencies were recorded for each question, and descriptive statistics were calculated for pharmacist characteristics and for attitudes about the pharmacists’ role in harm reduction (Table 1). Respondents had to answer at least one question on the survey beyond the screening question to be included in analysis. Non-community pharmacists (e.g., hospitalists, those practicing in long-term care settings) were excluded from this analysis. Non-community pharmacists were excluded from the analysis to ensure that pharmacist opinion was assessed in respondents most likely to practice in patient-facing settings. Pharmacists practicing in inpatient facilities or long-term care environments have limited access to patient populations in need of needle exchange services.

**Table 1:** Kentucky community pharmacists’ characteristics (*n* = 827)

	*n* (%)
Terminal degree	
BSPharm	371 (44.9)
PharmD	445 (53.8)
Other degree (PhD, others)	9 (1.1)
No response	2 (0.2)
Years in practice	
0 to 5 years	203 (24.6)
6 to 10 years	124 (15)
11 to 20 years	157 (19)
> 20 years	341 (41)
No response	2 (0.2)
Pharmacist gender	
Female	417 (50.4)
Male	393 (47.5)
Prefer not to answer	17 (2.1)
Community pharmacy practice setting	
Chain or supermarket pharmacy	485 (58.6)
Independent pharmacy	342 (41.4)
Urban or rural practice setting	
Urban county	407 (49.2)
Rural county	395 (47.8)
County not provided	25 (3.0)

Two dependent variables were selected for multivariate analyses: community pharmacist willingness to provide clean needles/syringes and community pharmacist willingness to dispose of used needles/syringes. Both of the dependent variables were derived from Likert-type question items (e.g., “How willing are you to provide clean needles and syringes to injection drug users?” Response options included a scale from 1, which represents “not at all willing,” through 6, which represented “very willing.” The rank order nature of the dependent variables indicated use of ordinal regression, which was employed to estimate the impact of community pharmacist characteristics (e.g., pharmacist age, degree type, gender, years in practice, community pharmacy practice setting, whether they currently sell nonprescription needles/syringes, urban or rural practice setting, county of practice) and attitudes towards harm reduction on each of the dependent variables. Ordinality was comprised of the six response categories as they appeared on the survey instrument, where the reference category for both of the dependent variables is “1” (not at all willing). Alternative specifications of the model were created that limited analysis to only those community pharmacists who currently sell needles and syringes in their practice and limiting to only those who do not sell needles and syringes in their practice (see Additional file 2: Table S1). To understand how practice setting may influence syringe provision practices and willingness and attitudes toward harm reduction, several additional analyses considered differences by chain and supermarket community pharmacy setting.

Predictor and independent variables in the multivariate model were selected based on a backward elimination strategy from the model, where all demographic questions were included in the first model construction and variables were eliminated due to either multicollinearity (pharmacist age and pharmacist years in practice, with age eliminated), or poor model fit (pharmacist county of practice was re-coded as the better fitting “urban” or “rural” county). Pharmacist county of practice was transformed to “urban” or “rural” practice setting using the US Department of Agriculture Rural-Urban Continuum Codes, which classifies all counties based on population thresholds [24]. Model variables were tested for multicollinearity via the construction of a correlation matrix, resulting in elimination of the pharmacist age variable from the final model.

Two assessment items of pharmacist attitudes towards harm reduction were included on the survey instrument: “Pharmacists could have significant public health impact by providing access to syringes/needles for IV drug users,” and “Access to clean syringes/needles is important to prevent blood-borne infections such as HIV and hepatitis in IV drug users.” These items were constructed as Likert-type items, where respondents were asked to select a level of agreement with each statement on a scale of 1 to 6 (with 1 representing “strongly disagree” and 6 representing “strongly agree”). Responses to these attitude assessments were also included as predictor variables in the multivariate models, but responses of “Don’t know” for these questions were omitted from analysis.

Needle and syringe sales practices were summarized from survey questions about frequency of sales and refusal to sell nonprescription needles and syringes (i.e., “Do you sell syringes and needles without a prescription at your pharmacy?” [Yes or No]; “In the last 30 days, to how many individuals have you sold syringes and needles?”; “In the past 30 days, how many times have you denied requests for the purchase of syringes or needles?”). Responses about mean sales and mean sale refusals from chain and supermarket community pharmacists were compared against responses from independent community pharmacists using two-tailed Student’s *t* testing. Additionally, bivariate testing using Pearson chi-square analysis of willingness to sell nonprescription needles/syringes, willingness to provide clean needles/syringes, and willingness to dispose of used needles/syringes were conducted between independent community pharmacists and chain/supermarket community pharmacists who currently sell needles and syringes in their practice. This analysis was repeated for only those community pharmacists who currently do not sell needles and syringes in their practice.

Pharmacists provided free-text responses to the question, “What are the barriers to selling syringes or needles without a prescription at your pharmacy?”. Community pharmacist-reported barriers to selling syringes and needles were analyzed for common themes, and the frequency of occurrence for each barrier theme was counted for all community pharmacists, and then by practice setting. Responses of “Don’t Know,” “Unsure,” “NA,” and “None,” were not included in the analysis. Differences in the frequency of each barrier theme reported between independent pharmacists and chain/supermarket pharmacists were compared statistically using Pearson chi-square analysis. A priori significance was set at 0.05, and all statistical analyses were conducted in Stata v13.0 [25].

## Results

An email survey invitation was successfully delivered to 4699 Kentucky pharmacists and 1282 completed the survey (response rate of 27.28%). Only community pharmacists were included in analysis (*n* = 827). Nearly 60% of respondents practiced in chain or supermarket community pharmacies, and more than half of respondents had been in practice for over 10 years. Distribution of pharmacists between urban and rural practice settings was evenly split.

**Fig. 1:**
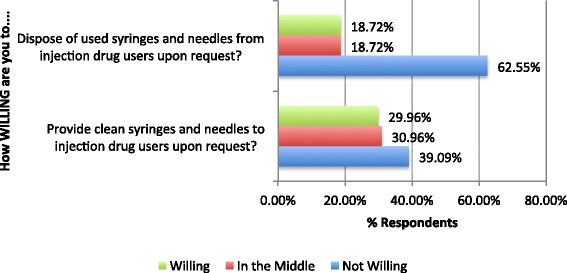
Community pharmacists’ willingness to participate in needle exchange (*n* = 705). Wilingness scores were collapsed for clarity (1 and 2 = not willing, 3 and 4 = in the middle, 5 and 6 = willing). Responses of “Don’t Know” are not included on the figure

**Fig. 2:**
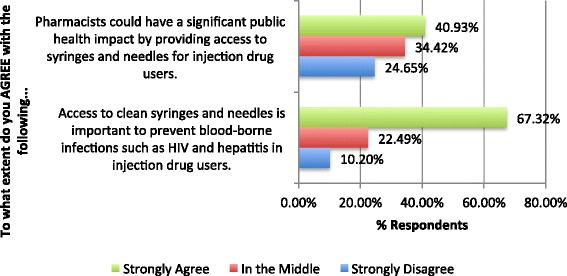
Community pharmacists’ attitudes towards harm reduction (*n* = 716). Agreement scores were collapsed for clarity (1 and 2 = strongly disagree, 3 and 4 = in the middle, 5 and 6 = strongly agree). Responses of “Don’t Know” are not included on the figure

Pharmacists were divided on willingness to provide clean needles and syringes, with 39.1% not willing (score 1 or 2 of 6) and 30% willing (score 5 or 6 of 6). Few pharmacists expressed willingness to dispose of used needles and syringes, with 62.6% not willing (score 1 or 2 of 6) and only 18.7% willing (score 5 or 6 of 6). Figure 1 shows responses for willingness to participate in NEP activities while Fig. 2 provides a distribution of responses for pharmacists’ attitudes towards harm reduction strategies.

Community pharmacists were divided about the statement, “Pharmacists could have a significant public health impact by providing access to syringes and needles for injection drug users,” with about a third of respondents unable to agree or disagree (Fig. 2). More than two thirds of community pharmacists strongly agree that “access to clean syringes and needles is important to prevent blood-borne infections such as HIV and hepatitis” in PWIDs (67.3%, 5 or 6 of 6) and with only 10.2% of community pharmacists strongly disagreeing with that statement (score 1 or 2 of 6).

Table 2 provides results from the multivariate analyses. Community pharmacists who expressed agreement with the statement that pharmacists could have significant public health impact by providing access to clean syringes/needles for PWID were more likely to express willingness to provide clean needles and syringes (OR 3.56; 95% CI 3.06–4.15) and were also more likely to express willingness to dispose of used needles and syringes (OR 2.04; 95% CI 1.77–2.35).

**Table 2:** Community pharmacists’ willingness to participate in needle exchange activities, ordinal logistic regression analysis results

	Willingness to provide clean needles and syringes^2^ (*n* = 628)		Willingness to dispose of used needles and syringes^2^ (*n* = 632)	
	Adjusted odds ratio	95% CI	Adjusted odds ratio	95% CI
Terminal degree				
BSPharm	Ref.		Ref.	
PharmD	1.00	0.60–1.67	1.40	0.82–2.40
Years in practice				
0 to 5 years	Ref.		Ref.	
6 to 10 years	0.76	0.46–1.25	0.77	0.46–1.29
11 to 20 years	1.30	0.82–2.08	1.48	0.91–2.42
> 20 years	0.93	0.50–1.70	1.51	0.80–2.82
Pharmacist gender				
Female	0.74	0.54–1.01	0.72*	0.52–0.99
Male	Ref.		Ref.	
Community pharmacy practice setting				
Chain or supermarket pharmacy	0.92	0.65–1.30	0.61*	0.43–0.87
Independent pharmacy	Ref.		Ref.	
Urban or rural practice setting				
Urban county	1.04	0.75–1.44	0.96	0.69–1.33
Rural county	Ref.		Ref.	
Sells needles/syringes without a prescription				
Yes, sells needles/syringes	1.19	0.85–1.65	0.92	0.66–1.29
No, does not sell needles/syringes	Ref.		Ref.	
Pharmacist attitudes^1^				
Pharmacists could have significant public health impact by providing access to syringes/needles for IV drug users	3.56*	3.06–4.15	2.04*	1.77–2.35
Access to clean syringes/needles is important to prevent blood-borne infections such as HIV and hepatitis in IV drug users	1.04	0.91–1.20	0.91	0.78–1.06

^1^Respondents could select a response on a scale of 1 (strongly disagree) to 6 (strongly agree) for each attitude question. Responses of “Don’t Know” were not included in regression analysis

^2^The reference group for the dependent variable in both willingness models is a response of 1 (Not at all willing)

*Indicates statistical significance

Community pharmacist agreement with the statement that clean syringes/needles are important for blood-borne infection prevention was not a significant predictor of pharmacist willingness to provide clean needles/syringes nor to dispose of used needles/syringes. Community pharmacists in chain or supermarket pharmacy practice settings were 39% less likely to express willingness to dispose of used needles/syringes (95% CI 0.43–0.87) when compared with community pharmacists in independent practice settings. Female pharmacists were 28% less likely to express willingness to dispose of used needles/syringes than male pharmacists (95% CI 0.52–0.99). No other pharmacist characteristics measured in this survey were associated with willingness to provide clean needles/syringes or willingness to dispose of used needles/syringes.

Additional file 2: Table S1 provides results for two alternative specifications of the multivariate models, where only those community pharmacists who currently sell needles and syringes were included in alternative specification A, and those who do not sell needles and syringes were included in alternative specification B. Results were similar in the alternative specifications to the results presented in Table 2, with the only significant covariate for both willingness to provide clean needles and willingness to dispose of used needles found among those who agreed that pharmacists could have a significant public health impact by providing access to syringes/needles for IV drug users and who currently sell needles/syringes (willingness to provide clean needles aOR 3.82, 95% CI 3.11–4.69; willingness to dispose of used needles aOR 2.25, 95% CI 1.84–2.75). Pharmacist agreement with having a significant public health impact remained the only significant covariate for willingness to provide clean needles among pharmacists who do not currently sell needles/syringes (aOR 3.34; 95% CI 2.64–4.24). In the model that incorporated those who do not currently sell syringes/needles, only female gender and practicing in a chain/supermarket pharmacy were significant factors in willingness to dispose of used needles (female aOR 0.47, 95% CI 0.27–0.84; chain/supermarket aOR 0.39, 95% CI 0.21–0.73)

Table 3 summarizes needle and syringe sales practices among community pharmacists, with chain and supermarket pharmacists compared against independent community pharmacists. More than a third of community pharmacists reported that they currently do not sell syringes/needles without a prescription at their pharmacies (36.7% do not sell, 63.3% will sell), with significantly fewer independent community pharmacists selling needles/syringes without a prescription than chain/supermarket community pharmacists (51.9% independent pharmacists sell; 71.5% chain/supermarket community pharmacists sell; *p* < 0.01). Chain/supermarket community pharmacists, on average, also report selling more syringes and needles than independent pharmacists (*p* = 0.01); however, chain/supermarket community pharmacists also report denying requests for syringes/needles more often than their independent community pharmacist counterparts (*p* < 0.01).

**Table 3:** Community pharmacists’ self-reported needle and syringe sales practices, independent pharmacists vs. chain/supermarket pharmacists

Needle/syringe sales practice	All community pharmacists	Independent pharmacists	Chain/supermarket pharmacists	Independent vs. chain/supermarket^1^
	*N* (%)	*N* (%)	*N* (%)	*P* value
Do you sell needles and syringes without a prescription in your pharmacy?				
Yes	445 (63.3)	152 (51.9)	293 (71.5)	< 0.01*
No	258 (36.7)	141 (48.1)	117 (28.5)	
Among all community pharmacists: “How willing are you to provide clean needles and syringes?”				
Willing (5 or 6)	210 (30.0)	100 (33.8)	110 (27.2)	
In the middle (3 or 4)	217 (31.0)	85 (28.7)	132 (32.6)	0.04*
Not at all willing (1 or 2)	274 (39.1)	111 (37.5)	163 (40.2)	
Among all community pharmacists: “How willing are you to dispose of used needles and syringes?”				
Willing (5 or 6)	132 (18.7)	70 (23.7)	62 (15.2)	
In the middle (3 or 4)	132 (18.7)	60 (20.3)	72 (17.6)	< 0.01*
Not at all willing (1 or 2)	441 (62.6)	166 (56.1)	275 (67.2)	
Only those who do not sell needles: “How willing are you to provide clean needles and syringes?”				
Willing (5 or 6)	50 (20.41)	34 (25.19)	16 (14.55)	
In the middle (3 or 4)	74 (30.20)	40 (29.63)	34 (30.91)	0.08
Not at all willing (1 or 2)	121 (49.39)	61 (45.19)	60 (54.55)	
Only those who do not sell needles: “How willing are you to dispose of used needles and syringes?”				
Willing (5 or 6)	41 (16.33)	29 (21.01)	12 (10.62)	
In the middle (3 or 4)	48 (19.12)	32 (23.19)	16 (14.16)	< 0.01*
Not at all willing (1 or 2)	162 (64.54)	77 (55.80)	85 (75.22)	
	Mean (SD)	Mean (SD)	Mean (SD)	*P* value
In the last 30 days, to how many individuals have you sold syringes and needles?	10.29 (30.4)	4.79 (7.4)	13.20 (36.9)	0.01*
In the past 30 days, how many times have you DENIED requests for purchase of syringes or needles?	2.51 (4.9)	1.36 (4.5)	3.12 (5.0)	< 0.01*

^1^Differences in response frequencies for independent vs. chain/supermarket pharmacist responses were compared via chi-square analysis for the question, “Do you sell needles and syringes without a prescription in your pharmacy?” Willingness to provide and dispose of needles and syringes between independent and chain/supermarket pharmacists were also compared via chi-square analysis. All other comparisons between independent and chain/supermarket pharmacists were conducted via means testing (two-tailed *t* tests)

*Indicates statistical significance

Independent pharmacists were, on average, more willing to provide clean needles/syringes (*p* = 0.04) as well as more likely to dispose of used needles/syringes (*p* < 0.01) when compared with their chain/supermarket pharmacist counterparts. Among all community pharmacists, a majority (62.6%) expressed unwillingness to dispose of used syringes and needles (score 1 or 2 of 6) but fewer (39.1%) were unwilling to provide clean needles and syringes (score 1 or 2 of 6). Among community pharmacists who do not currently sell needles, independent pharmacists were more likely to report willingness to dispose of used needles and syringes than chain/supermarket pharmacists (*p* < 0.01), but no statistically significant difference was observed for willingness to provide clean needles and syringes between independent and chain/supermarket pharmacists who currently do not sell needles/syringes (*p* = 0.08).

Table 4 summarizes the most frequently occurring responses to the open response question, “What are the barriers to selling syringes or needles without a prescription at your pharmacy?” A total of *n* = 172 community pharmacists (20.8% of community pharmacist respondents) reported at least one barrier to selling syringes or needles, and *n* = 655 community pharmacists did not articulate a barrier. Overall, most respondents who reported facing a barrier to selling syringes or needles in their pharmacies indicated that ethical concerns about supplying materials for abuse or illegitimate use was a significant barrier (*n* = 71, or 41.3% of all community pharmacist respondents), followed by concerns about the clientele who would patronize the pharmacy (*n* = 44, or 25.6% of all community pharmacist respondents).

**Table 4:** Frequently reported barriers to selling syringes/needles without a prescription, as reported by Kentucky community pharmacists

Theme of reported barrier	All community pharmacists (*n* = 172)^1^	Independent pharmacists (*n* = 85)	Chain/supermarket pharmacists (*n* = 87)	Independent vs. chain/supermarket
	*n*	*n* (%)	*n* (%)	*p* value
Concern about clientele in the pharmacy	44	11 (25.0)	33 (75.0)	< 0.01*
Ethical concerns about supplying materials for abuse or illegitimate use	71	40 (56.3)	31 (43.7)	0.13
Conflict with city ordinance or company policy	25	13 (52.0)	12 (48.0)	0.78
Other legal concerns	7	3 (42.9)	4 (57.1)	0.72
Problems with record-keeping	22	16 (72.7)	6 (27.3)	0.02*
Time	6	3 (50.0)	3 (50.0)	0.98
Reputation with colleagues or community	5	2 (40.0)	3 (60.0)	0.67
Concern of finding or handling used needles	6	0 (0.00)	6 (100.00)	0.01*
Supply problems	3	1 (33.3)	2 (66.7)	0.57

^1^Respondent may have written more than one comment for this question, so totals do not sum to 100%

*Indicates statistical significance

Chain/supermarket pharmacists reported barriers related to clientele concerns significantly more frequently than independent pharmacists (*p* < 0.01), as well as more frequent barriers related to concern about finding or having to handle used needles when compared with independent pharmacists (*p* = 0.01). Independent pharmacists reported barriers related to problems with record keeping practices significantly more frequently than chain/supermarket pharmacists (*p* = 0.02). This suggests that there are different barriers to selling needles and syringes perceived by independent versus chain/supermarket pharmacists.

## Discussion

Kentucky community pharmacists in any practice setting expressed more willingness to provide clean needles and syringes than to dispose of used needles and syringes. This mirrors results from a recent evaluation of patient experiences with pharmacy-based NEPs in China which found that disposal of used needles was the most problematic element [26]. Only 63.3% of surveyed community pharmacists in Kentucky currently permit sales of needles/syringes without a prescription despite state law that authorizes non-prescription sales. The findings of this study in regard to the attitudes related to the provision and disposal of needles and syringes suggest that efforts to promote NEPs in Kentucky may more successfully target independent community pharmacists based on expressed reluctance from chain and supermarket community pharmacists; however, community pharmacists in either setting are better positioned to provide convenient access to these types of services when compared to pharmacists in other practice settings (e.g., hospitalists, long-term care). It should be noted, however, that there is room for improvement among pharmacy-based protocols to address safety precautions as well as attitudes towards syringe provision and disposal among all community pharmacist groups. Interestingly, few differences were found among opinions between pharmacists practicing in rural and urban settings.

NEPs offered through fixed-location pharmacies attract a different demographic mix of patients when compared to mobile exchange service sites operating in the same area, [27] which suggests that different venues will attract different subsets of those who need access to NEPs. Promising pilot programs have also demonstrated that NEPs offered through pharmacies have been able to link clients to medical and social services more successfully than traditional exchange program sites [28]. Considering that community pharmacists reported significant barriers to selling nonprescription needles and syringes based on their concerns about ethics and clientele, the findings from this study suggest that substantial pharmacist education and outreach are needed. To address issues with workflow, the removal of requirements for recording syringe and needle purchases as well as standardization of protocol and process for syringe/needle storage, disposal, billing, physical location for placement, and co-messaging for syringe/needle disposal containment are warranted.

The American Pharmacists Association (APhA) recently published a call for community pharmacists to engage in harm reduction and public health-driven activities to address the opioid epidemic, with specific mention of supervised injection sites and the selling of nonprescription needles and syringes [29]. APhA and other professional organizations have the opportunity to engage with community pharmacists by expanding access to continuing education interventions and promoting the adoption of clear workflow guidelines and protocols by chain and supermarket community pharmacies, whose pharmacists demonstrated more reluctance to participate in NEP services in this survey when compared with independent community pharmacists. Additionally, universities and schools that train pharmacists should update and modify public health curriculum by including educational modules on harm reduction opportunities and responsibilities for pharmacists in community practice settings. Kentucky in particular may benefit from a harm reduction public health tool like NEPs when considering that surveillance systems from the Centers for Disease Control and Prevention report that the highest rates of acute hepatitis B and C infections in the USA were found in Kentucky from 2010 through 2013 [30].

This study has several limitations. First, it is possible that nonresponse bias from the pharmacists who did not choose to participate influenced findings. The response rate is low compared to traditional mail surveys, though this was found to be within the typical range observed in contemporary studies employing electronically delivered surveys [31]. Also, it is possible (and perhaps likely) that the attitudes and opinions of Kentucky pharmacists are not generalizable to or representative of pharmacists in other states or countries. Additionally, Kentucky may have a greater proportion of independent pharmacies than is typically found in other states, which may have contributed to the ability to detect differences by practice setting and conclusions related to harm reduction efforts with independent pharmacies and may limit generalizability of our findings to locations with different pharmacy setting characteristics.

## Conclusions

Kentucky community pharmacists report uncertainty about their role in harm reduction and NEPs in particular; however, they mostly agree that they do have a role to play in public health prevention efforts. Community pharmacists report significant barriers to selling/providing nonprescription needles and syringes and report even larger barriers for disposing of used needles and syringes in their pharmacies. Pharmacists practicing in independently owned pharmacies in Kentucky appear to be more receptive to selling than pharmacists working in chain or supermarket pharmacies, though pharmacists in chain practice settings currently report selling needles and syringes at a greater volume than their independent pharmacist counterparts.

## Additional file

Additional file 1: Survey Instrument. (PDF 73 kb)
Additional file 2: Table S1. Willingness to participate in needle exchange activities among community pharmacists who do or do not currently sell needles and syringes, ordinal logistic regression analysis results. (DOCX 15 kb)

## Abbreviations

BOP: Board of pharmacy
CI: Confidence interval
HIV: Human immunodeficiency virus
IV: Intravenous
NEP: Needle exchange program
OR: Odds ratio
PWID: People who inject drugs
REDCap: Research electronic data capture
SD: Standard deviation
