# Supplementary material for: Pharmacists’ role in harm reduction: a survey assessment of Kentucky community pharmacists’ willingness to participate in syringe/needle exchange
Source: Harm Reduct J. 2018 Jan 25;15:4. doi: 10.1186/s12954-018-0211-4 (PMC5785823; doi:10.1186/s12954-018-0211-4)
Supplement: Additional file 1: — Survey Instrument. (PDF 73 kb) [file 12954_2018_211_MOESM1_ESM.pdf]

Pharmacists' Role in Opioid Overdose Prevention

Please complete the survey below.

Thank you!

COVER LETTER FOR SURVEY

TITLE OF STUDY: Pharmacists' Role in Opioid Overdose Prevention

INVESTIGATOR INFORMATION: Patricia Freeman, PhD Phone: 859-323-1381

You are invited to participate in a survey collecting information and opinions related to the pharmacists role in opioid overdose prevention being conducted by the Center for the Advancement of Pharmacy Practice at the University of Kentucky College of Pharmacy in collaboration with the Kentucky Board of Pharmacy.

You have been asked to participate in this survey because you are a pharmacist licensed to practice in Kentucky. If you voluntarily complete the survey, you will be one of approximately 4500 pharmacists to do so. The survey will take approximately 10 minutes to complete.

The survey asks about your knowledge of opioid overdose and use of naloxone for overdose prevention, your opinions of the pharmacist's role in overdose prevention, and general demographic information. The information generated from this research will assist with the development of programs designed to assist pharmacists in implementing naloxone dispensing and opioid overdose prevention programs.

Your response to the survey is anonymous. Neither the researchers nor the Board of Pharmacy will know who did, or did not, respond to the survey. The research team will not attempt to trace responses back to individuals. There are no known risks associated with disclosure of your opinions about opioid overdose prevention programs.

Please be aware, while we make every effort to safeguard your data once received on our servers via REDCap, given the nature of online surveys, as with anything involving the Internet, we can never guarantee the confidentiality of the data while still en route to us.

You may receive two additional email invitations to participate in this survey over the next two weeks if you did not initially respond to the survey. If you elect not to respond to the survey, please ignore these additional emails.

Taking part in this research is completely voluntary. If you choose not to participate, there will be no penalty to you. You are free to skip any question that you do not want to answer and you can discontinue the survey at any time. Although you will not personally benefit by completing the survey the information that you provide may help with the implementation of opioid overdose prevention programs by pharmacists.

This study has been reviewed by the University of Kentucky Medical Institutional Review Board. If you have questions about this study, you may call Patricia Freeman at 859-323-1381. If you have any questions about your rights as a volunteer in this research, you may contact the staff in the Office of Research Integrity at the University of Kentucky at 859-257-9428 or toll free at 1-866-400-9428.

Thank you for your time and we appreciate your consideration in completing this survey.

Sincerely,

Patricia Freeman, PhD
Associate Professor
University of Kentucky College of Pharmacy

Do you currently practice pharmacy in the Commonwealth of Kentucky?

◯ Yes
◯ No

Thank you for your time and interest. The survey is designed for pharmacists who currently practice in Kentucky.

Section I: Pharmacist Characteristics

My age in years is:

- ◯ 21-25
- ◯ 26-34
- ◯ 35-44
- ◯ 45-64
- ◯ 65+

My terminal degree is:

- ◯ BSPharm
- ◯ PharmD
- ◯ MSPharm
- ◯ PhD
- ◯ Other terminal degree (please describe):

Please describe: ________________________________

My gender is:

- ◯ Male
- ◯ Female
- ◯ Prefer not to answer

My total number of years in practice, including residency:

- ◯ 0-2 years
- ◯ 3-5 years
- ◯ 6-10 years
- ◯ 11-20 years
- ◯ greater than 20 years

The practice setting where I spend most of my time practicing is best described as:

- ◯ Independent community pharmacy
- ◯ Chain community pharmacy
- ◯ Supermarket/mass merchandiser pharmacy
- ◯ Hospital pharmacy
- ◯ Ambulatory clinic
- ◯ Long-term care pharmacy
- ◯ Other, please describe

What type of hospital pharmacy?

- ◯ Inpatient
- ◯ Outpatient

Do you provide emergency department pharmacy services?

- ◯ Yes
- ◯ No

Do you dispense from your clinic?

- ◯ Yes
- ◯ No

Do you provide long-term care dispensing services?

- ◯ Yes
- ◯ No

Please describe: ________________________________

What are the number of hours you work in a pharmacy per week on average?

- ◯ less than 8 hours
- ◯ 8-23 hours
- ◯ 24-36 hours
- ◯ greater than 36 hours

What is the Kentucky county where you currently practice? (If you practice in more than one county, select the county where you practice most often.)

- ◯ Adair
- ◯ Allen
- ◯ Anderson
- ◯ Ballard
- ◯ Barren
- ◯ Bath
- ◯ Bell
- ◯ Boone
- ◯ Bourbon
- ◯ Boyd
- ◯ Boyle
- ◯ Bracken
- ◯ Breathitt
- ◯ Breckenridge
- ◯ Bullitt
- ◯ Butler
- ◯ Caldwell
- ◯ Calloway
- ◯ Campbell
- ◯ Carlisle
- ◯ Carroll
- ◯ Carter
- ◯ Casey
- ◯ Christian
- ◯ Clark
- ◯ Clay
- ◯ Clinton
- ◯ Crittenden
- ◯ Cumberland
- ◯ Daviess
- ◯ Edmonson
- ◯ Elliot
- ◯ Estill
- ◯ Fayette
- ◯ Fleming
- ◯ Floyd
- ◯ Franklin
- ◯ Fulton
- ◯ Gallatin
- ◯ Garrard
- ◯ Grant
- ◯ Graves
- ◯ Grayson
- ◯ Green
- ◯ Greenup
- ◯ Hancock
- ◯ Hardin
- ◯ Harlan
- ◯ Harrison
- ◯ Hart
- ◯ Henderson
- ◯ Henry
- ◯ Hickman
- ◯ Hopkins
- ◯ Jackson
- ◯ Jefferson
- ◯ Jessamine
- ◯ Johnson
- ◯ Kenton
- ◯ Knott
- ◯ Knox
- ◯ Larue
- ◯ Laurel
- ◯ Lawrence
- ◯ Lee
- ◯ Leslie
- ◯ Letcher
- ◯ Lewis
- ◯ Lincoln
- ◯ Livingston

◯ Logan
◯ Lyon
◯ Madison
◯ Magoffin
◯ Marion
◯ Marshall
◯ Martin
◯ Mason
◯ McLean
◯ McCracken
◯ McCreary
◯ Meade
◯ Menifee
◯ Mercer
◯ Metcalfe
◯ Monroe
◯ Montgomery
◯ Morgan
◯ Muhlenberg
◯ Nelson
◯ Nicholas
◯ Ohio
◯ Oldham
◯ Owen
◯ Owsley
◯ Pendleton
◯ Perry
◯ Pike
◯ Powell
◯ Pulaski
◯ Robertson
◯ Rockcastle
◯ Rowan
◯ Russell
◯ Scott
◯ Shelby
◯ Simpson
◯ Spencer
◯ Taylor
◯ Todd
◯ Trigg
◯ Trimble
◯ Union
◯ Warren
◯ Washington
◯ Wayne
◯ Webster
◯ Whitley
◯ Wolfe
◯ Woodford

What is the zip code of the Kentucky address where you currently practice? (If you practice at more than one address, select the zip code where you practice most often.)

____
(Zip code)

Section II: Opioid Overdose Prevention

How many times have you ever responded to an incident of respiratory depression caused by (or suspected to be caused by) opioids?

____
(enter number above)

Where were you when you responded to an incident of respiratory depression? (check all that apply)

- ☐ In a community setting
- ☐ In a hospital setting
- ☐ In a pharmacy setting
- ☐ In a school setting
- ☐ Other, please describe

Please describe: __________________________________

Have you ever administered naloxone to a person experiencing respiratory depression caused by (or suspected to be caused by) opioids?

- ◯ Yes
- ◯ No

Have you ever dispensed naloxone for an individual to take home and use for opioid overdose prevention?

- ◯ Yes
- ◯ No

In which of the following situations have you dispensed naloxone for an individual to take home and use for opioid overdose prevention? (Check all that apply.)

- ☐ An individual with a history of opioid overdose
- ☐ An individual who had a high dose opioid prescription (>100mg equivalents morphine daily)
- ☐ An individual who was opioid naïve and received a methadone prescription
- ☐ An individual who received an opioid prescription but had a history of smoking, COPD, respiratory illness or obstruction
- ☐ An individual who received an opioid prescription but had a history of renal dysfunction or hepatic disease
- ☐ An individual who had a concurrent benzodiazepine prescription
- ☐ An individual who had known or suspected alcohol abuse
- ☐ Other (please specify)
- ☐ None of these
- ☐ I don't know

Please specify __________________________________

What percent of patients to whom you dispensed naloxone did you provide education on the use of naloxone for overdose prevention?

- ◯ 10 percent
- ◯ 25 percent
- ◯ 50 percent
- ◯ More than 50 precent
- ◯ I provided no patient education
- ◯ I don't know

What percent of patients to whom you dispensed naloxone had already received education elsewhere on overdose prevention and naloxone use (i.e.. from a prescriber, clinic, community organization or family member)?

- ◯ 10 percent
- ◯ 25 percent
- ◯ 50 percent
- ◯ More than 50 percent
- ◯ I don't know

How many times have you dispensed naloxone to an indivdual in the past 30 days?

____
(enter number above)

Section III: Naloxone Access Programs

Have you ever been trained in overdose prevention and naloxone administration?

◯ Yes
◯ No

What was the name of the training program you attended?

____

Do you plan to apply for certification in Kentucky to initiate the dispensing of naloxone via a prescriber-approved protocol?

◯ Yes
◯ No

When do you plan to apply for certification?

◯ I have already applied for certification
◯ I will apply in the next 6 months
◯ I will apply within 6-12 months
◯ I will apply in 1-2 years
◯ I am not sure

How CONFIDENT are you in your ability to:

	1 Not at all confident	2	3	4	5	6 Extremely confident	Don't know
Identify signs and symptoms of opioid overdose	◯	◯	◯	◯	◯	◯	◯
Proactively identify individuals who may be at risk for opioid overdose and would benefit from a naloxone prescription	◯	◯	◯	◯	◯	◯	◯
Educate patients to recognize opioid overdose and safely administer naloxone when indicated	◯	◯	◯	◯	◯	◯	◯

How WILLING are you to:

	1 Not willing at all	2	3	4	5	6 Very much willing	Don't know
Attend an education and training program on opioid overdose prevention	○	○	○	○	○	○	○
Enter into a protocal agreement with a physician authorizing the initiation of naloxone dispensing	○	○	○	○	○	○	○
Proactively identify individuals meeting criteria for naloxone dispensation under a protocol	○	○	○	○	○	○	○
Initiate the dispensing of naloxone under a protocol agreement with a physician	○	○	○	○	○	○	○
Dispense naloxone for opioid overdose prevention with a valid prescription	○	○	○	○	○	○	○
Educate patients to recognize opioid overdose and safely administer naloxone when indicated	○	○	○	○	○	○	○

Which of the following are barriers that you foresee in implementing a naloxone access program at your practice? (Check all that apply)

- ☐ Time to develop and implement program
- ☐ Support from manager or administration for program
- ☐ Knowledge regarding the law and regulations authorizing naloxone access programs
- ☐ Complications with billing and reimbursement
- ☐ Moral or ethical concerns associated with drug abuse and overdose
- ☐ Concerns over clientele that might frequent the pharmacy if a program were in place
- ☐ Packaging or stocking the various forms of naloxone
- ☐ Community opposition to a local naloxone access program
- ☐ Other barriers
- ☐ None of these are barriers

Please describe

____

Which of the barriers you selected above represents the most significant challenge to implementing a naloxone access program at your practice?

- ○ Time to develop and implement program
- ○ Support from manager or administration for program
- ○ Knowledge regarding the law and regulations authorizing access programs
- ○ Complications with billing and reimbursement
- ○ Moral or ethical concerns associated with drug abuse and overdose
- ○ Concerns over clientele that might frequent the pharmacy if a program were in place
- ○ Packaging or stocking the various forms of naloxone
- ○ Community opposition to a local naloxone access program
- ○ Other barrier as listed above

Section IV: Attitudes Toward Overdose Prevention

Below are a series of statements. Please choose the number corresponding to your agreement with each statement. There are no right or wrong answers. We are interested in hearing your opinions about these statements.

	1 Strongly disagree	2	3	4	5	6 Strongly agree	Don't know
Overdose prevention for people who use opioids is a waste of time and money	○	○	○	○	○	○	○
Training people how to identify and respond to an overdose sends the message that misusing pain medications is OK	○	○	○	○	○	○	○
Pharmacists have a role to play in injury prevention, including overdose prevention	○	○	○	○	○	○	○
Overdose prevention programs encourage misuse of opioids	○	○	○	○	○	○	○

Please indicate whether this sentence is true, false or don't know: Good Samaritan laws shield individuals who call 911 to help someone experiencing an overdose from being charged or prosecuted with possession of narcotics or controlled substances (including pain medication) if these substances are found at the scene of the event.

○ True
○ False
○ Don't know

Please indicate whether this sentence is true, false or don't know: Kentucky currently has a Good Samaritan law as described above.

○ True
○ False
○ Don't know

Section V: Syringe and Needle Access

Do you sell syringes and needles without a prescription at your pharmacy?
◯ Yes
◯ No

In the last 30 days, to how many individuals have you sold syringes and needles?
____
(enter number here)

In the past 30 days, how many times have you denied requests for purchase of syringes or needles?
____
(enter number here)

Why did you deny a request for the purchase of the syringes or needles?
____

In the past 30 days, how many requests for syringes or needles have you denied?
____
(enter number above)

What are the barriers to selling syringes or needles without a prescription at your pharmacy?
____

Below are a series of statements. Please choose the number corresponding to your agreement with each statement. There are no right or wrong answers. We are interested in hearing your opinions about these statements.

To what extent do you AGREE with following statements:

	1 Strongly disagree	2	3	4	5	6 Strongly agree	Don't know
Access to clean syringes and needles is important to prevent blood-borne infections such as HIV and hepatitis in injection drug users.	◯	◯	◯	◯	◯	◯	◯
Pharmacists could have a significant public health impact by providing access to syringes and needles for injection drug users.	◯	◯	◯	◯	◯	◯	◯

Assuming state law allowed it and mechanisms for reimbursement were in place, how willing would you be to:

	1 Not willing at all	2	3	4	5	6 Very much willing	Don't know
Provide clean syringes and needles to injection drug users upon request?	◯	◯	◯	◯	◯	◯	◯
Dispose of used syringes and needles from injection drug users upon request?	◯	◯	◯	◯	◯	◯	◯

We welcome any comments or feedback from you.
____
