# Supplementary material for: Pharmacists’ role in harm reduction: a survey assessment of Kentucky community pharmacists’ willingness to participate in syringe/needle exchange
Source: Harm Reduct J. 2018 Jan 25;15:4. doi: 10.1186/s12954-018-0211-4 (PMC5785823; doi:10.1186/s12954-018-0211-4)
Supplement: Additional file 2: Table S1. — Willingness to participate in needle exchange activities among community pharmacists who do or do not currently sell needles and syringes, ordinal logistic regression analysis results. (DOCX 15 kb) [file 12954_2018_211_MOESM2_ESM.docx]

Additional file 2: Table S1: Willingness to Participate in Needle Exchange Activities Among Community Pharmacists Who Do or Do Not Currently Sell Needles and Syringes, Ordinal Logistic Regression Analysis Results

|  | Specification A: Those who Sell Needles/Syringes | | | | Specification B: Those who do NOT Sell Needles/Syringes | | | |
| --- | --- | --- | --- | --- | --- | --- | --- | --- |
|  | Willingness to Provide Clean Needles and Syringes^2^ (n=402) | | Willingness to Dispose of Used Needles and Syringes^2^ (n=402) | | Willingness to Provide Clean Needles and Syringes^2^ (n=226) | | Willingness to Dispose of Used Needles and Syringes^2^ (n=230) | |
|  | *aOR* | *95% CI* | *aOR* | *95% CI* | *aOR* | *95% CI* | *aOR* | *95% CI* |
| Terminal Degree |  |  |  |  |  |  |  |  |
| BSPharm | Ref. |  | Ref. |  | Ref. |  | Ref. |  |
| PharmD | 1.10 | 0.58-2.10 | 1.34 | 0.67-2.65 | 0.84 | 0.70-1.98 | 1.66 | 0.15-4.03 |
| Years in Practice |  |  |  |  |  |  |  |  |
| 0 to 5 years | Ref. |  | Ref. |  | Ref. |  | Ref. |  |
| 6 to 10 years | 0.64 | 0.36-1.16 | 0.95 | 0.52-1.72 | 1.11 | 0.42-2.93 | 0.43 | 0.15-1.22 |
| 11 to 20 years | 1.29 | 0.73-2.27 | 1.54 | 0.84-2.84 | 1.30 | 0.54-3.16 | 1.47 | 0.62-3.51 |
| >20 years | 1.02 | 0.48-1,16 | 1.69 | 0.77-3.70 | 0.78 | 0.27-2.26 | 1.16 | 0.40-3.38 |
| Pharmacist Gender |  |  |  |  |  |  |  |  |
| Female | 0.72 | 0.49-1.06 | 0.90 | 0.61-1.34 | 0.78 | 0.45-1.37 | 0.47* | 0.27-0.84 |
| Male | Ref. |  | Ref. |  | Ref. |  | Ref. |  |
| Community Pharmacy Practice Setting |  |  |  |  |  |  |  |  |
| Chain or Supermarket Pharmacy | 0.96 | 0.63-1.47 | 0.73 | 0.47-1.12 | 0.79 | 0.43-1.45 | 0.39* | 0.21-0.73 |
| Independent Pharmacy | Ref. |  | Ref. |  | Ref. |  | Ref. |  |
| Urban or Rural Practice Setting |  |  |  |  |  |  |  |  |
| Urban County | 0.96 | 0.65-1.44 | 0.91 | 0.61-1.33 | 1.19 | 0.67-2.12 | 1.23 | 0.68-2.23 |
| Rural County | Ref. |  | Ref. |  | Ref. |  | Ref. |  |
| Pharmacist Attitudes^1^ |  |  |  |  |  |  |  |  |
| Pharmacists could have significant public health impact by providing access to syringes/needles for IV drug users | 3.82* | 3.11-4.69 | 2.25* | 1.84-2.75 | 3.34* | 2.64-4.24 | 1.85* | 1.49-2.29 |
| Access to clean syringes/needles is important to prevent blood-borne infections such as HIV and hepatitis in IV drug users | 0.95 | 0.76-1.15 | 0.82 | 0.65-1.02 | 1.15 | 0.94-1.40 | 1.00 | 0.81-1.25 |

*^1^Respondents could select a response on a scale of 1 (Strongly disagree) to 6 (Strongly agree) for each attitude question. Responses of “Don’t Know” were not included in regression analysis.*

*^2^The reference group for the dependent variable in both willingness models is a response of 1 (Not at all willing).*

**Indicates statistical significance.*
